# Evaluating the QEAS-7 questionnaire as a predictor of asbestos burden in lung tissue among lung cancer patients: insights from the AMCANES study

**DOI:** 10.3389/ftox.2026.1733214

**Published:** 2026-03-06

**Authors:** Galo Granados, Antía Ferreiro, Carlos Gómez-Ollés, Francisco-Javier González-Barcala, Annie Navarro, Roser Costa, Fernando Romero, Belén Marín, María E. Barroso, Isabel Urrutia, Sandra Dorado-Arenas, Ane Uranga Echeverría, José-María Marín, Larraitz Garcia, Sara Calero, Mayte Martín-Bustamante, Coral Márquez, María-Jesús Cruz, Jaume Ferrer

**Affiliations:** 1 Hospital Universitari Vall d'Hebron/Vall d'Hebron Institut de Recerca, Barcelona, Spain; 2 Department of Respiratory Medicine, Universitat Autònoma de Barcelona (UAB), Barcelona, Spain; 3 Institut Català de Seguretat i Salut Laboral, Barcelona, Spain; 4 Respiratory Department, Complejo Hospitalario Universitario de Santiago de Compostela, Santiago de Compostela, Spain; 5 Translational Research in Airway Diseases (TRIAD), Fundación Instituto de Investigación Sanitaria de Santiago de Compostela (FIDIS), Santiago de Compostela, Spain; 6 Department of Medicine. University of Santiago de Compostela, Santiago de Compostela, Spain; 7 Hospital Sant Joan de Déu, Martorell, Spain; 8 Althaia Xarxa Assistencial Universitària de Manresa, Manresa, Spain; 9 Institut de Recerca i Innovació en Ciències de la Vida i de la Salut a la Catalunya Central (IRIS-CC), Vic, Spain; 10 Hospital Universitario Puerta del Mar, Cádiz, Spain; 11 Hospital Universitario de Navarra, Pamplona, Spain; 12 Hospital General Universitario Dr. Balmis, Alicante, Spain; 13 Hospital Universitario Galdakao-Usansolo, Galdakao, Spain; 14 Instituto de Investigación Sanitaria Biobizkaia, Galdakao, Spain; 15 Hospital Universitario Miguel Servet, Zaragoza, Spain; 16 Hospital Universitario de Cruces, Barakaldo, Spain; 17 Hospital Universitario de Getafe, Getafe, Spain; 18 CIBER Enfermedades Respiratorias (CIBERES), Instituto de Salud Carlos III, Madrid, Spain

**Keywords:** asbestos, cancer, diagnosis, exposure, questionnaire

## Abstract

**Objectives:**

Asbestos exposure is a known risk factor for lung cancer, and quantifying asbestos bodies (ABs) in lung tissue remains the gold standard for assessing cumulative exposure. However, histological analysis is not routinely available in many clinical settings. The validated QEAS-7 questionnaire was developed to evaluate occupational, domestic, and environmental asbestos exposure. This study examined the association between QEAS-7 exposure classifications and the asbestos burden in lung cancer patients.

**Methods:**

A cross-sectional multicenter study was conducted across nine Spanish hospitals. Patients with histologically confirmed lung cancer undergoing surgical resection completed the QEAS-7 questionnaire. Non-tumoral lung tissue samples were analyzed for Abs and asbestos burden was categorized according to AB concentration (with elevated burden defined as >1000 AB/g). Diagnostic performance metrics were evaluated.

**Results:**

Among 133 patients (median age 67 years; 77% male), ABs were detected in 61% of samples, with 5% showing an elevated asbestos burden (>1000 AB/g). Occupational exposure was reported by 36% of patients and accounted for 71.4% of cases with elevated burden. Domestic exposure was reported by 9% of patients and detected in 28.6% of elevated-burden cases. The combined occupational and domestic exposure domain achieved 100% sensitivity for detecting elevated asbestos burden, while occupational and domestic domains alone showed sensitivities of 71.4% and 28.6%, respectively. Specificity and positive predictive values remained modest. Notably, 29% of patients classified as ‘non-exposed’ by QEAS-7 had measurable ABs.

**Conclusion:**

The QEAS-7 questionnaire demonstrated high sensitivity for detecting elevated asbestos burden when combining occupational and domestic exposures. While histological analysis remains the definitive method, QEAS-7 offers a practical screening alternative, when lacking access to tissue-based diagnostics or occupational hygiene expertise.

## Introduction

Asbestos is a fibrous mineral widely used in industrial applications due to its excellent thermal insulation, fire resistance, and resistance to corrosion (Frank and Joshi, 2014). It is classified into two main groups: serpentines, such as chrysotile, characterized by their curved and fragmentable fibers, and amphiboles, including crocidolite and amosite, which have straight, rigid, and chemically resistant fibers (Gunter, 2010; Gualtieri, 2023). When inhaled, both types can penetrate deep into the lungs, bypass the body’s natural clearance mechanisms, and provoke chronic inflammation (Furuya et al., 2018; Ruosaari et al., 2008; Mossman et al., 1998).

Asbestos exposure is associated with a spectrum of respiratory conditions, ranging from benign pleural abnormalities to severe diseases such as asbestosis, pleural mesothelioma, and lung cancer (American Thoracic Society, 2004; Spagnolo et al., 2023; van et al., 2024; Doniach et al., 1975). Epidemiological studies consistently demonstrate a higher risk of mesothelioma and lung cancer among asbestos-exposed workers, especially in trades such as construction and shipbuilding (Markowitz, 2015; Berman and Crump, 2008a; Huh et al., 2024). The carcinogenicity of asbestos fibers is attributed to their resistance to biological degradation, which enables them to persist in lung tissue and induce chronic inflammation, oxidative stress, and genetic mutations that contribute to cancer development (Gamble and Gibbs, 2008; Rasmuson et al., 2014).

Quantifying asbestos bodies (ABs) in lung tissue is essential for both diagnostic and medico-legal evaluations. A concentration exceeding 1,000 ABs per gram of dry lung tissue is generally considered indicative of significant past exposure (Feder et al., 2017; Ai, 2004). However, this measurement is technically challenging, requires specialized histopathological analysis, and is not routinely available in all clinical settings. Moreover, despite the well-documented health risks associated with asbestos, early identification of exposed individuals remains problematic due to the limited sensitivity and accuracy of current exposure assessment tools. While Job Exposure Matrices provide useful estimates of occupational exposure and support epidemiological research, they do not capture all exposure scenarios and often lack individual-level specificity (Ahrens et al., 1993; Pintos et al., 2009).

Moreover, although structured questionnaires are commonly used to assess asbestos exposure, their interpretation requires specialized knowledge in occupational hygiene. Many studies also lack validation data and clear evaluation criteria. Furthermore, conventional tools often fail to detect non-occupational exposures, such as environmental contamination or secondary household exposure (Feder et al., 2017; Siemiatycki et al., 2004; Wolff et al., 2015; Dumortier et al., 2001; Damiran and Frank, 2023). The QEAS-7 questionnaire is a structured tool designed to assess an individual’s potential exposure to asbestos and has been validated for routine clinical use (Ferrer et al., 2020).

This study aims to explore the relationship between asbestos exposure—assessed using the QEAS-7 questionnaire—and the presence of asbestos bodies (ABs) in lung tissue among patients with histologically confirmed lung cancer, in order to evaluate the questionnaire’s effectiveness as a reliable screening instrument.

## Methods

### Study design and setting

This was a multicenter, cross-sectional observational study that included patients with a confirmed diagnosis of lung cancer who consecutively underwent pulmonary resection surgery—either pneumonectomy or lobectomy. A total of 133 patients were recruited from nine tertiary care hospitals across Spain: Complejo Hospitalario de Navarra (Pamplona), Hospital Universitario de Santiago de Compostela (Santiago de Compostela), Hospital Miguel Servet (Zaragoza), Hospital General Universitario Dr. Balmis (Alicante), Hospital Universitario Puerta del Mar (Cádiz), Hospital Mútua Terrassa (Barcelona), Hospital de Cruces (Bilbao), Hospital de Galdakao (Bilbao), and Hospital General de Getafe (Madrid).

These centers ensured geographic and demographic representation from several autonomous communities, including Andalusia, Aragon, Catalonia, Galicia, Madrid, Navarra, the Basque Country, and the Valencian Community. The study was conducted under the framework of the AMCANES project (*Asbestos in Malignant Cancers of the Lung: National Epidemiologic Study*), approved by the respective Research Ethics Committees of all participating institutions and carried out in accordance with the principles of the Declaration of Helsinki.

### Clinical study

Patients were recruited from dedicated lung cancer outpatient clinics at participating centers. A clinical evaluation was conducted, including a review of medical history, demographic data (age, sex), detailed smoking history (type of tobacco, onset, cessation, duration, and pack-years), and personal or family history of lung diseases. Radiological images with CT scans were reviewed. Pathological confirmation of lung cancer was performed at each center following standard histological protocols using hematoxylin-eosin staining.

### Questionnaire (QEAS-7)

Asbestos exposure was assessed during the first follow-up visit using the original QEAS-7 (Asbestos Exposure Assessment Questionnaire), a validated instrument designed to evaluate exposure through three distinct pathways: occupational, domestic, and environmental (Ferrer et al., 2020). The questionnaire comprises seven items—three addressing occupational exposure, two domestic, and two environmental—supplemented by two reference lists: one with 48 occupations/activities and another with 70 asbestos-containing materials (ACMs), each color-coded by exposure risk—red for high risk and black for moderate risk.

Participants provided a chronological occupational history and identified any relevant exposures from the provided lists, including estimated exposure duration. Domestic exposure was assessed by inquiring about cohabitation with individuals occupationally exposed to asbestos or contact with contaminated objects. Environmental exposure was evaluated based on proximity to asbestos-related industries or contaminated areas.

All questionnaires were independently reviewed by trained industrial hygienists. Based on questionnaire responses, occupational exposure was classified into four categories: certain, probable, non-existent, or unknown, considering general and specific responses, the type of exposure source, and its associated risk level. This structured approach allowed for a comprehensive yet efficient evaluation of asbestos exposure across multiple contexts, ensuring consistent classification and methodological rigor.

### Occupational exposure intensity assessment

In addition to estimating the probability of occupational asbestos exposure, intensity was assessed using a semi-quantitative algorithm developed in the AMCANES study (Institut Català de Seguretat i Salut Laboral, 2023). This method considered three variables: the time period of exposure, the estimated airborne fiber concentration (f/cc), and the duration of exposure. Exposure periods were stratified into three historical intervals: Before 1981 (10–100 f/cc), 1982–2002 (1–10 f/cc), Post-2002 (0.1–1 f/cc), to reflect major shifts in industrial practices, asbestos use, and regulatory measures in Spain that influenced workplace fiber concentrations. Defining these intervals allows cumulative exposure assessments to account not only for total duration but also for temporal variations in exposure intensity, providing a more accurate estimation of occupational asbestos burden across different historical periods. Exposure durations were classified as: >15 years, 5–15 years, <5 years. An intensity score ranging from 1 (Very Low) to 5 (Very High) was assigned based on the combination of these factors. For individuals with multiple exposures, the highest probability value prevailed in the final estimation.

For analytical purposes, hospitals were grouped based on regional asbestos exposure risk. Group 1 included centers located in historically high-risk industrial areas (e.g., shipbuilding, construction), while Group 2 comprised hospitals from regions with lower documented asbestos exposure. This classification allowed for exploration of geographic variation in pulmonary asbestos burden among lung cancer patients.

### Asbestos body measurement

Asbestos body (AB) quantification was performed as previously described (De et al., 1998). Briefly, lung tissue samples (2 cm^3^) were obtained from non-tumorous areas located at least 5 cm from the tumor margin. Two 0.5 g fragments were prepared per sample, carefully avoiding pleura and blood vessels.

One fragment was frozen and lyophilized to determine dry weight, enabling standardized quantification of ABs per gram of dry lung tissue. The second fragment was subjected to chemical digestion with 30 mL of filtered sodium hypochlorite under constant agitation for 24 h to eliminate organic material.

Following digestion, samples were centrifuged at 3,700 rpm for 20 min, resuspended in filtered distilled water, and treated ultrasonically (UCI-50, 300W, 50/60Hz; Raypa S.L.) for 10 min. The suspensions were then washed and filtered through a 0.45 µm Millipore membrane (HAWP02500). Filters were dried at 37 °C overnight and clarified using an acetone vaporizer (JSHoldings, 240V/110V) prior to microscopic examination.

Asbestos bodies were counted under a phase-contrast microscope (Optiphot) at ×500 magnification. Only structures ≥5 µm in length were included. Results were expressed as the number of asbestos bodies per gram of dry lung tissue.

For analysis, patients were stratified into three categories based on AB burden: low (0–300 AB/g), intermediate (301–999 AB/g), and high (>1000 AB/g). The high-burden threshold (>1000 AB/g) was selected based on precedent from previous studies (De et al., 1998), while the low- and intermediate-burden cut-points were defined according to the distribution of ABs in our cohort, allowing separation of the majority of patients with very low asbestos exposure from those with higher exposures.

### Statistical analysis

Descriptive statistics were used to summarize the study population. Categorical variables were expressed as absolute frequencies and percentages. Continuous variables were presented as mean and standard deviation (SD) or median and interquartile range (IQR), according to data distribution assessed using the Shapiro–Wilk test. Associations between clinical and exposure-related variables and the asbestos body burden in lung tissue were evaluated using non-parametric tests. The Mann–Whitney U test or Kruskal–Wallis test was applied for comparisons of continuous variables, and the Chi-squared test or Fisher’s exact test for categorical variables. The correlation between the QEAS-7 questionnaire score and the number of asbestos bodies per gram of dry lung tissue was analyzed using Spearman’s rank correlation coefficient. A p-value <0.05 was considered statistically significant in all tests. Statistical analysis was performed using R version 4.3.2 (R Foundation for Statistical Computing, Vienna, Austria).

## Results

A total of 133 patients with histologically confirmed lung cancer were included in the analysis. The median age was 67 years (IQR 61–72), and 77% were male. Regarding smoking history, 29% were active smokers, 61% former smokers, and 10% had never smoked. In terms of occupational background, 54% of the study population had worked in sectors historically associated with asbestos exposure, such as the construction, metallurgical, and naval industries, as well as mechanics, painters, drivers, the textile sector, and office roles in asbestos-exposed environments (Table 1).

**TABLE 1:** Demographic characteristics of the study population.

Data	AB/g dry tissue				
	0–300	301–999	>1,000	Total	p-value
	n = 111	n = 15	n = 7	n = 133	
Age, median (IQR), years	66 (37–83)	68 (58–79)	65 (51–86)	67 (61–72)	0.441
Gender, male, n (%)	84 (75%)	12 (80%)	7 (100)	103 (77)	0.939
Smoking habit, n (%)	​	​	​	​	0.8501
Non smoker	13 (12)	1 (7)	0 (0)	14 (10)	​
Smoker	32 (29)	4 (27)	2 (29)	38 (29)	​
Former smoker	66 (59)	10 (66)	5 (71)	81 (61)	​
Smoker, paq/yr, median (IQR)	42 (7–120)	31 (15–98)	97 (50–144)	50.3 (30)	0.1953
Charlson index, median (IQR)	5 (2–11)	5 (4–7)	5 (4–10)	5 (1.7)	0.3737
Pneumological history n (%)					
COPD	2 (29)	10 (67)	30 (27)	42 (32)	0.014
Asthma	4 (4)	0 (0)	0 (0)	4 (3)	NS
OSA	6 (5)	0 (0)	0 (0)	6 (5)	NS
Respiratory infections	9 (8)	1 (6.7)	0 (0)	10 (8)	NS
Work industry, n (%)					
Food industry	6 (86)	1 (14)	0 (0)	7 (5)	0.6333
Construction industry	21 (88)	1 (4)	2 (8)	24 (18)	​
Metallurgical y naval industry	9 (64)	5 (36)	0 (0.0)	14 (11)	​
Textile industry	2 (67)	0 (0.0)	1 (33)	3 (2)	​
Office work in asbestos-exposed industries	4 (57)	2 (29)	1 (14)	7 (5)	​
Drivers	4 (50)	1 (13)	3 (37)	8 (6)	​
Mechanics and painters	14 (87)	2 (12)	0 (0)	16 (12)	​
Chemicals industry	2 (100)	0 (0)	0 (0)	2 (1)	​
Others	49 (94)	3 (6)	0 (0)	52 (39)	​
AB/g dry tissue, median (IQR)	46 (0–272)	463 (341–787)	1,475 (1,064–12980)	​	​

AB, asbestos bodies; COPD, chronic obstructive pulmonary disease; OSA, obstructive sleep apnea; IQR , interquartile range.

Asbestos bodies (ABs) were detected in 81 (61%) of lung tissue samples. Most patients 111 (84%) fell into the lowest burden category (0–300 AB/g dry tissue), whereas 15 (11%) were in the intermediate range (301–999 AB/g), and 7 (5%) exceeded the high-burden threshold (>1000 AB/g). The median asbestos burden in the highest group was 1475 AB/g (IQR: 1,064–12980), compared to 46 AB/g (IQR: 0–272) in the lowest group. Tumor location was most frequently observed in the left upper lobe (32%) and right upper lobe (29%). Fifty-nine percent of patients had adenocarcinoma, 29% squamous cell carcinoma, and 12% presented with other histological types (Table 2). No statistically significant differences in asbestos burden were observed across histological subtypes of lung cancer (p = 0.395).

**TABLE 2:** Cancer-related clinical features in the study population.

Data n (%)	AB/gr dry tissue				
	0–300	301–999	>1,000	Total	p-value
	n = 111	n = 15	n = 7	n = 133	
Chest CT scan location					
Right upper lobe	30 (27)	5 (33)	3 (43)	38 (29)	0.432
Middle lobe	12 (10)	4 (27)	0 (0)	16 (12)	​
Right lower lobe	13 (12)	2 (13)	1 (14)	16 (12)	​
Left upper lobe	36 (32)	4 (27)	2 (29)	42 (32)	​
Left lower lobe	20 (18)	0 (0)	1 (14)	21 (16)	​
Cancer stage					
IA	38 (35)	5 (33)	1 (14)	44 (34)	0.529
IB	12 (11)	5 (33)	1 (14)	18 (14)	​
IIA	13 (12)	0 (0)	1 (14)	14 (11)	​
IIB	19 (17)	3 (20)	3 (43)	25 (19)	​
IIIA	20 (18)	2 (13)	1 (14)	23 (18)	​
IIIB	3 (3)	0 (0)	0 (0)	3 (2)	​
IVA	4 (4)	0 (0)	0 (0)	4 (3)	​
Histopathological diagnosis*					
Adenocarcinoma	65 (58)	7 (47)	7 (100)	79 (59)	0.395
Squamous cell	31 (23)	7 (47)	0 (0)	38 (29)	​
Small cell	0 (0)	0 (0)	4 (4)	4 (3)	​
Neuroendocrine	0 (0)	0 (0)	6 (5)	6 (5)	​
Metastasis from other organs	0 (0)	1 (7)	4 (57)	5 (4)	​

AB, asbestos bodies; CT, computed tomography; IA–IVA, tumor stages according to the TNM, Staging System that includes the extent of the tumor (T), extent of spread to the lymph nodes (N), and presence of metastasis (M): * Some patients presented more than one histopathological diagnosis.

The association between asbestos burden and exposure classification according to the QEAS-7 questionnaire is summarized in Table 3. A total of 48 patients (36%) reported ‘certain’ occupational exposure, representing 71.4% (5 out of 7) of those with an asbestos burden exceeding 1000 AB/g. Likewise, ‘certain’ domestic exposure was reported by 12 patients (9%), including 28.6% (2 out of 7) of those with high asbestos burden. Notably, no patients with AB >1,000 reported ‘certain’ environmental exposure. Occupational exposure intensity, categorized as very low, low, intermediate, high, or very high, showed no significant association with asbestos burden across groups (p = 0.893). Notably, 26 patients with high or very high occupational intensity were found in the group with AB <300 AB/g, suggesting that intensity estimates did not consistently predict elevated tissue burden. Patients from Group 1 hospitals, located in historically high-risk industrial areas, accounted for 86% of the high ABs burden group (p = 0.021), indicating relevant regional differences in asbestos exposure.

**TABLE 3:** Validity of the exposure questionnaire in detecting asbestos exposure (QEAS-7).

QEAS-7 questionnaire n (%)	AB/gr dry tissue				
	0–300	301–999	>1,000	Total	p-value
Occupational exposure	n = 82	n = 13	n = 5	n = 100	​
No exposure	37 (45)	4 (31)	0 (0)	41 (41)	0.1483
Probably	10 (12)	1 (8)	0 (0)	11 (11)	​
Certain	35 (43)	8 (61)	5 (100)	48 (48)	​
Domestic exposure	n = 63	n = 5	n = 2	n = 70	​
No exposure	37 (59)	4 (80)	0 (0)	41 (59)	0.0793
Probably	16 (25)	1 (20)	0 (0)	17 (24)	​
Certain	10 (16)	0 (0)	2 (100)	12 (17)	​
Environmental exposure	n = 40	n = 5	n = 0	n = 45	​
No exposure	37 (92)	4 (80)	0 (NaN)	41 (91)	0.133
Probably	0 (0)	1 (20)	0 (NaN)	1 (2)	​
Certain	3 (8)	0 (0)	0 (NaN)	3 (7)	​
Occupational intensity	n = 45	n = 9	n = 5	n = 59	​
Very low	3 (7)	0 (0)	0 (0)	3 (5)	0.893
Low	7 (16)	0 (0)	1 (20)	8 (14)	​
Intermediate	9 (20)	3 (33)	1 (20)	13 (22)	​
High	12 (27)	2 (23)	2 (40)	16 (27)	​
Very high	14 (30)	4 (44)	1 (20)	19 (32)	​
Patient’s origin	n = 111	n = 15	n = 7	n = 133	​
Group 1	55 (49)	12 (80)	6 (86)	73 (55)	0.021
Group 2	56 (50)	3 (20)	1 (14)	60 (45)	​

AB, asbestos bodies; QEAS-7, questionnaire for the evaluation of asbestos exposure; Group 1, hospitals located in historically high-exposure regions; Group 2, hospitals in lower-risk areas. Patients with both occupational and domestic exposure were classified as occupational. Domestic and environmental groups include only exclusive exposures.

**FIGURE 1:**
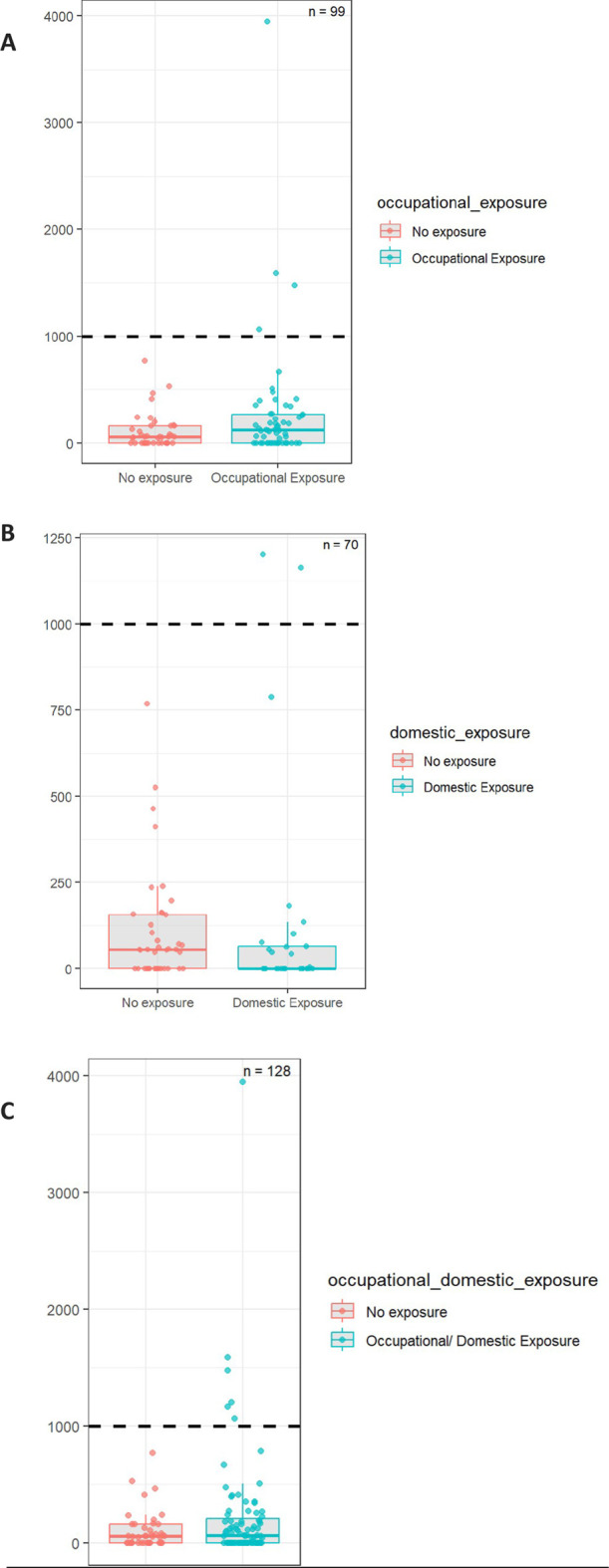
Boxplot and scatterplot illustrating the distribution of asbestos bodies per gram of dry lung tissue (AB/g) by exposure group **(A)** occupational, **(B)** domestic, **(C)** combined).

Table 4 summarizes the diagnostic performance of the QEAS-7 questionnaire evaluated separately for each exposure domain. Sensitivity for detecting elevated asbestos burden (>1000 AB/g) was 71.4% for the occupational dimension, 28.6% for the domestic dimension, and 100% for the combined occupational and domestic exposure. Specificity was 43.2% for occupational exposure, 60.3% for domestic exposure, and 33.6% for combined exposure. Positive predictive values (PPV) were 8.5% for occupational, 6.9% for domestic and 8.0% for combined exposure. Negative predictive values (NPV) were 97.6% for occupational, 95.5% for domestic and 100% for combined exposure. The positive likelihood ratio (LR+) ranged from 0.72 (domestic) to 1.51 (combined), while the negative likelihood ratio (LR–) ranged from 0.66 (occupational) to 1.19 (domestic); LR–was 0 for the combined exposure domain. Figure 1 presents a box-and-whisker plot with overlaid scatterplot illustrating the distribution of asbestos body burden (AB/g) across the exposure categories. The dashed line represents the predefined diagnostic threshold of 1000 AB/g.

**TABLE 4:** Diagnostic performance of the QEAS-7 questionnaire in predicting elevated asbestos burden (>1000 AB/g) by exposure domain.

Exposure domain	Sensitivity (%; IC)	Specificity (%; IC)	PPV	NPV	LR+	LR-
Occupational	71.4 (29.0; 96.3)	43.2 (33.0; 53.7)	8.5 (2.8; 18.7)	97.6 (91.4; 99.7)	1.26 (0.96; 1.64)	0.66 (0.16; 2.69)
Domestic	28.6 (3.7; 70.9)	60.3 (47.7; 72.0)	6.9 (0.9; 22.8)	95.5 (88.7; 98.8)	0.72 (0.41; 1.27)	1.19 (0.69; 2.05)
Occupational + domestic	100 (59.04; 100)	33.61 (25.31; 42.72)	7.95 (3.26; 15.7)	100 (91.4; 100)	1.51 (1.33; 1.71)	0 (0; NaN)

IC, 95% confidence interval; PPV, positive predictive value; NPV, negative predictive value; LR+, positive likelihood ratio; LR–, negative likelihood ratio; NaN, not a number.

## Discussion

This cross-sectional multicenter study (AMCANES) demonstrated the feasibility and clinical value of the QEAS-7 questionnaire in estimating pulmonary asbestos body (AB) burden among patients with histologically confirmed lung cancer. By integrating standardized histological analysis with patient-reported exposure data across occupational, domestic, and environmental domains, the study identified meaningful associations between reported exposure and AB burden. These findings support the potential role of QEAS-7 as a practical screening tool in clinical contexts where tissue-based diagnostics may not be readily available.

In our cohort, asbestos bodies were detected in 61% of lung tissue samples, yet only 5% of patients exceeded the diagnostic threshold of 1000 AB/g. This prevalence aligns with necropsy-based studies in European populations, where elevated asbestos burden ranged from 4% to 6% (Doniach et al., 1975; Karjalainen et al., 1996). Similarly, an autopsy study in Spain reported detectable asbestos bodies in 83% of an urban population, although only 3% exceeded 1000 AB/g, underscoring the relevance of this threshold as a marker of significant exposure (Velasco-et al., 2010). These findings suggest that even in populations with documented exposure, high fiber retention is not universal, potentially influenced by individual clearance mechanisms and fiber type (e.g., amphiboles vs. chrysotile).

When evaluating the diagnostic performance of the QEAS-7 by exposure dimension, only the combined occupational and domestic exposure achieved 100% sensitivity (95% CI: 59.04–100) for detecting elevated asbestos burden, while occupational and domestic exposures alone yielded sensitivity of 71.4% and 28.6%, respectively. These findings align with prior validation studies of the QEAS-7, which showed high sensitivity when integrating multiple exposure sources (Ferrer et al., 2020). Retrospective case–control studies have similarly highlighted the value of detailed occupational histories for assessing asbestos-related lung cancer risk, and pooled analyses such as the SYNERGY project confirmed a strong association between occupational asbestos exposure and lung cancer, particularly with co-exposure to silica or welding fumes (Ahrens et al., 1993). While those studies used expert-reviewed job-exposure matrices, our findings suggest that patient-reported tools like QEAS-7 questionnaire may offer complementary value, especially in settings without occupational health expertise. Although the QEAS-7 has been primarily validated for lung cancer risk assessment, it could also have substantial value in the evaluation of non-malignant asbestos-related lung diseases, such as pulmonary fibrosis, particularly in the absence of pleural plaques. Future studies should explore its application in this context to support clinical differentiation and exposure assessment.

In addition to expert-reviewed job-exposure matrices and other research questionnaires, structured tools have been developed to capture detailed asbestos exposure histories. For example, Gaitens et al. (2023) describe a comprehensive patient exposure questionnaire for mesothelioma registries, which collects extensive occupational, residential, and hobby-related exposure data. While such tools are valuable for epidemiological research, the QEAS-7 was specifically designed as a brief, clinically feasible screening instrument, facilitating rapid identification of individuals likely to have significant asbestos exposure in routine practice. This brevity and simplicity provide advantages in clinical contexts where time and occupational hygiene expertise are limited, complementing more detailed research-oriented questionnaires.

Notably, 29% of patients classified as ‘non-exposed’ by the QEAS-7 were found to have measurable asbestos bodies in lung tissue, a discrepancy greater than that reported in more homogeneous autopsy-based cohorts (Doniach et al., 1975; Velasco et al., 2010), likely reflecting underreporting or unrecognized exposure. It is important to emphasize that QEAS-7 questionnaire is an integrated screening tool, where ‘positive’ exposure is defined by reporting certain exposure in any domain. Therefore, its diagnostic evaluation should reflect this combined interpretation, consistent with its role as a unified screening instrument. While separate analysis of individual dimensions may offer exploratory insights, it does not align with its diagnostic purpose. Accordingly, global sensitivity and specificity from combined domains represent its true diagnostic performance, as supported by prior validation studies (Ferrer et al., 2020).

No significant association was found between occupational exposure intensity and asbestos burden. Although 26 patients were classified with high or very high occupational intensity, most had low asbestos burden (<300 AB/g). This discrepancy highlights the limitations of retrospective intensity estimates in predicting tissue burden, likely influenced by fiber type (e.g., chrysotile vs. amphiboles), latency, or clearance variability (Berman and Crump, 2008b; Velasco et al., 2017; Roggli et al., 2002). Only amphibole fibers were retained in lung tissue, supporting the hypothesis of preferential clearance of chrysotile (Berman and Crump, 2008b). Similarly, most mesothelioma cases in the United States were associated with elevated tissue levels of commercial amphiboles, with minimal chrysotile retention, emphasizing the predominant role of amphiboles in asbestos-related disease (Roggli et al., 2002; Rödelsperger et al., 1999). Although less biopersistent, chrysotile has also been associated with increased risks of mesothelioma and lung cancer, challenging claims of its relative safety (Stayner et al., 1996).

Beyond detecting high asbestos burden (>1000 AB/g), QEAS-7 questionnaire also identified patients with intermediate (301–999 AB/g) and low (<300 AB/g) burden across occupational and domestic exposures. This underscores its ability to capture a broad exposure spectrum, including low-intensity or historical exposures with epidemiological relevance. Notably, 86% of those reporting ‘certain’ occupational exposure had low asbestos burden; similar patterns were observed for domestic exposure. These findings reaffirm the influence of fiber type, latency, and clearance on tissue burden (Velasco-et al., 2010; Berman and Crump, 2008b).

The domestic exposure dimension identified 28.6% of patients with a high asbestos burden (>1000 AB/g), underscoring its relevance in detecting secondary exposures, such as household contact with contaminated work clothes or residential proximity to asbestos-related industries (Magnani et al., 2000). Incorporating this dimension improves the detection of non-occupational cases that might be overlooked if relying solely on occupational history.

Although no patients with >1000 AB/g reported ‘certain’ environmental exposure, the environmental domain identified individuals with low or intermediate asbestos burden. This suggests that while environmental exposure may contribute to some degree of asbestos fiber retention, its impact is limited compared to occupational or domestic sources. Consequently, environmental exposure appears to play a marginal role in the development of high-burden cases and offers limited diagnostic utility. Similar findings have been reported in studies of prolonged environmental exposure (Krówczyńska and Wilk, 2019; Tamura et al., 2018).

Regional variability was evident, with 86% of patients with high asbestos burden coming from hospitals in historically high-risk industrial areas, reflecting known geographic disparities in asbestos-related disease (Magnani et al., 2000; Bellis et al., 2024). No significant differences in asbestos burden were found across histological subtypes or tumor stages, supporting the role of asbestos as a co-carcinogen rather than a determinant of tumor type (Gamble and Gibbs, 2008; Rasmuson et al., 2014). The multicenter design strengthens the study, as the significant regional differences observed (p = 0.021) highlight how local industrial history influences individual exposure risk, consistent with previous reports from Mediterranean and European industrial regions (Magnani et al., 2000; Bellis et al., 2024).

The relationship between self-reported exposure and asbestos body burden in this study partially aligns with previous validation research showing a positive correlation between re30 29). A large validation study found linear associations between estimated exposure and fiber concentrations measured by light and electron microscopy, emphasizing the value of exposure modeling even without direct environmental measurements (Rasmuson et al., 2014). Notably, chrysotile fibers are less reliably detected in lung tissue due to lower biopersistence (Berman and Crump, 2008b; Velasco et al., 2017), which may explain variability in asbestos body counts, especially among patients classified as ‘non-exposed’ by the QEAS-7 but with measurable asbestos bodies. These findings underscore the need to consider both fiber type and exposure intensity when interpreting asbestos body loads and support the complementary role of validated clinical questionnaires alongside conventional exposure-reconstruction methods.

Although this multicenter study included a geographically diverse cohort, several limitations should be noted. First, this study focuses exclusively on lung cancer and not on mesothelioma; asbestos body quantification was used as a marker of cumulative exposure, which is relevant for lung cancer risk assessment, whereas fibre characteristics such as aspect ratio—important in mesothelioma pathogenesis—were beyond the scope of this study and were not evaluated. Second, no additional biomarkers were measured, including biomarkers of lung inflammation, as the study was focused specifically on assessing the association between QEAS-7 exposure classifications and asbestos body burden in lung tissue. Moreover, due to the cross-sectional design and inclusion of only lung cancer patients, it is not possible to define a burden threshold above which the risk of lung cancer increases. In addition, the small number of patients with high asbestos burden (>1000 AB/g) limited subgroup analyses. Reliance on self-reported exposure may have introduced recall bias, as evidenced by measurable asbestos bodies in 29% of patients classified as ‘non-exposed.’ Furthermore, histological analysis may underestimate total fiber burden by excluding uncoated fibers. Although it is theoretically possible to back-calculate asbestos body burden into estimates of average workplace exposure, our study was not designed for quantitative reconstruction of occupational exposure levels. Future studies combining detailed occupational history, industrial hygiene data, and tissue burden measurements could address this question and provide more precise exposure–response estimates. Finally, further research is needed to validate the QEAS-7 questionnaire in other populations and to assess complementary diagnostic methods.

In conclusion, the QEAS-7 questionnaire is a sensitive and practical screening tool for identifying lung cancer patients at risk of elevated asbestos burden, particularly when combining occupational and domestic exposures. Although it does not directly quantify asbestos bodies, its correlation with histological findings supports its clinical utility, especially in settings without access to tissue analysis or occupational health expertise. Given its modest specificity, complementary diagnostic methods are recommended in medico-legal contexts. Further research in larger cohorts is needed to validate its role as a standardized screening instrument for asbestos-related diseases.

## Data Availability

The data supporting the findings of this study are available upon reasonable request. Due to ethical and privacy considerations, access is limited to ensure the confidentiality of patient information.
